# Effects of quasiperiodic forcing in epidemic models

**DOI:** 10.1063/1.4963174

**Published:** 2016-09-22

**Authors:** Shakir Bilal, Brajendra K. Singh, Awadhesh Prasad, Edwin Michael

**Affiliations:** 1 Department of Physics and Astrophysics, University of Delhi, Delhi 110 007, India; 2 Department of Biological Sciences, University of Notre Dame, Notre Dame, Indiana 46556, USA

## Abstract

We study changes in the bifurcations of seasonally driven compartmental epidemic models, where the transmission rate is modulated temporally. In the presence of periodic modulation of the transmission rate, the dynamics varies from periodic to chaotic. The route to chaos is typically through period doubling bifurcation. There are coexisting attractors for some sets of parameters. However in the presence of quasiperiodic modulation, tori are created in place of periodic orbits and chaos appears via finite torus doublings. Strange nonchaotic attractors (SNAs) are created at the boundary of chaotic and torus dynamics. Multistability is found to be reduced as a function of quasiperiodic modulation strength. It is argued that occurrence of SNAs gives an opportunity of asymptotic predictability of epidemic growth even when the underlying dynamics is strange.

Effects of seasonal variation in the spread of infectious diseases have been a subject of immense interest. The inclusion of seasonality in transmission models has helped in explaining spatiotemporal variations in incidence of infectious diseases such as measles. In general, seasonality is modelled by considering periodic modulation of the transmission rate in epidemic models. The periodic modulation was largely inspired by the fact that spread of measles in school going children is governed by the opening and closing of schools. Seasonality can be more complicated than just being periodic. For example, non-periodic fluctuations (e.g., quasiperiodic ones) in temperature and precipitation due to climate change may have complicated effects on disease incidence. Such non-periodic modulation in transmission presents the next level of complexity with respect to temporal variability in disease incidence. In this study, we examine the effects of quasiperiodic modulation in the transmission rate in compartmental epidemic models. A generic analysis of the parameter space across different epidemic models is also presented. We discuss the implication of the creation and coexistence of attractors on dynamical behaviour, such as strange nonchaotic attractors.

## I. INTRODUCTION

Seasonal variation in reported cases of infectious diseases, ranging from childhood diseases (e.g., measles, diphtheria, and chickenpox) to infections affecting all ages like influenza, cholera, and mosquito-borne diseases (e.g., malaria and dengue), is common across temperate and tropical geographies.^1–3^ Although there is a growing body of literature^1–6^ investigating the role of external drivers (e.g., rainfall, temperature, etc.) responsible for seasonal fluctuations in disease incidence, a better understanding of the contributions of external (climate- or environment-driven) versus internal (nonlinearity caused by immune responses in disease dynamics itself) factors to the observed seasonality is lacking and clearly forms an active area of research in the field of mathematical modelling and predictive epidemiology of infectious diseases.^6,7^

In typical seasonally forced models of infectious diseases, the transmission rate (i.e., the per capita rate at which a susceptible individual interacts with an infectious individual and acquires a new infection) is modulated in a periodic fashion using a sine or cosine function.^1–3,8,9^ (However, see Refs. 7 and 10 for an exception to this rule.) Although periodic forcing in the transmission term helps explain most of the observed seasonality in disease data, external drivers themselves may not be periodic or, at least, may not remain periodic over time.^6,11,12^ Instead, these external drivers may have anomalies in the amplitude and/or onset of the peaks that occur in different years. Such irregular dynamic behaviour cannot be captured by a simple sinusoidal function.^6^

In physics and engineering sciences, there have been extensive studies in forced oscillator systems. Both the nature of dynamics and the bifurcations in nonlinear dynamical systems are significantly modified in the presence of external forcing.^13,14^ If an unforced dynamical system is dissipative, different behaviours can result,^15,16^ and these issues have been investigated over the decades with particular focus on the different dynamical attractors that can be formed, the various transitions that take place, and their potential applications.^13,15,16^ In these studies, different types of forcing, most notably periodic, quasiperiodic, and random modulations, have been studied.

One of the characteristic features of temporally forced systems is that they possess multiple coexisting attractors: the dynamics sensitively depends on the initial values of state variables. However, when periodic forcing in these systems is replaced by quasiperiodic one (which introduces variability in the forcing strength because of irregularity in the amplitude and timing of the peak of the driving signal), the number of coexisting attracting states of these dissipative systems is reduced or the coexistence disappears altogether. Attracting states specific to quasiperiodically forced systems such as tori, strange nonchaotic attractors (SNAs) are also created. The dynamics on SNAs is characterised with no positive Lyapunov exponents but with an underlying fractal geometry (i.e., it is discontinuous everywhere).^15–17^

Coexistence of multiple attractors or states has also been observed in the dynamics of epidemic models when the transmission rates are periodically forced.^5,10,18,19^ Understanding the mechanisms behind the coexistence of multiple attractors in disease systems, and how the infection trajectories move in and out of the multiple attracting states, has important implications for control programmes.^5,19^ Although it is becoming increasingly apparent that the external drivers (e.g., rainfall, temperature, etc.) of seasonality do not vary in regular, periodic fashion,^6,11,12^ studies in epidemic models investigating the impact of temporal variability in the strength of the external drivers on outbreak patterns and its implication for control are scarce. Consequently, a better understanding of how internal factors (e.g., immunity) of disease dynamics, in association with variable (as opposed to regular/periodic) external forcing, shape up outbreak trajectories is lacking.

In this paper, therefore, we investigate the dynamics of epidemic models, varying in internal complexity, under quasiperiodic forcing of the transmission rate. We contrast the resultant behaviours (in terms of bifurcations and the coexistence of attractors) with those observed under periodic forcing. In doing so, we aim to get better insights about the combined role of external versus internal factors that they play in bringing about the seasonality seen in the incidences of infectious diseases. As hinted in the beginning of this paragraph, the models considered here vary in internal feedbacks on disease dynamics in terms of the effect of immune response in regulating the build-up of susceptibles during inter-epidemic periods necessary for fuelling next outbreaks. With quasiperiodic modulation, we capture the impact of the external feedback that may vary over time in its strength (i.e., amplitude) and/or the onset of the peak in the transmission term. The subsequent sections are organized as follows. In Section II, we describe a set of epidemic models with the temporally forced transmission rate. In the section, we also define the measures used to characterize SNA dynamics. In Section III, we discuss the results on the dynamics of the epidemics models with quasiperiodic modulation in the transmission rate vis-a-vis those obtained with periodic modulation. This is followed in Section IV by discussing the implications of our results.

## II. MODELS AND MEASURES

### A. Models

A typical epidemic model consists of the compartments of susceptibles (S), exposed (E), infectives (I), and recovered (R) individuals.^4,5^ At the start of an emerging infection outbreak, all individuals are susceptible to it; once infected they can remain dormant (exposed) for some time before infecting others or they immediately become infectives and start infecting others. The infectives meet one of the two outcomes: either they recover from and become immune to the infection or they succumb to the disease and die, and hence removed from the transmission chain. Additionally, we study the effect of human demographics (i.e., the birth and death rates) into the model dynamics. In our study, the newborns join the susceptible compartment, which means we do not consider vertical (i.e., mother to child) transmission, and the per capita births and deaths are taken to be the same to keep the population size constant, with the total host density scaled to 1.

• **SIR model**
  The SIR model consists of individuals of the susceptible, infective, and recovered classes. Susceptibles transition their state to become infectives who in turn move to the recovered class. Thus, the SIR model with demographics is given by the following equations:^5^
  $$\begin{array}{c} \dot{S}=\mu -\beta SI-\mu S,\\ \dot{I}=\beta SI-\eta I-\mu I,\\ \dot{R}=\eta I-\mu R, \end{array}$$ (1)
  where $\dot{X}$ represents the time derivative *dX*/*dt*, *β* the transmission rate, *μ* the human birth (or death) rate, and *η* the recovery rate.
• **SIRS model**
  Recovery from an infection may not provide a life-long immunity; the recovered individuals may become susceptible again. Such situations are modelled using the SIRS model given by^5^
  $$\begin{array}{c} \dot{S}=\mu -\beta SI-\mu S+\kappa R,\\ \dot{I}=\beta SI-\eta I-\mu I,\\ \dot{R}=\eta I-(\kappa +\mu )R, \end{array}$$ (2)
  where the parameters β,μ,η have the same meaning as in the SIR model while *κ* is the rate at which the recovered individuals become susceptible again. If κ→0 (i.e., recovered individuals do not become susceptible again), then the SIRS reduces to the SIR model.
• **SEIR model**
  Newly infected individuals of a population before they start infecting others may stay in an exposed stage. Such transmission process is studied using the SEIR model, and the model dynamics are given by the following equations:
  $$\begin{array}{c} \dot{S}=\mu -\beta SI-\mu S,\\ \dot{E}=\beta SI-\sigma E-\mu E,\\ \dot{I}=\sigma E-\eta I-\mu I,\\ \dot{R}=\eta I-\mu R, \end{array}$$ (3)
  where the parameters β,μ,η have the same meaning as in SIR model, while *σ* is the rate at which the exposed individuals join the infective class. It is clear that the SEIR model reduces to the SIR when σ→∞ (i.e., the latent period tends to zero).

The models of the SIR family described above differ from one another in the way the proportions of individuals progress through different infection stages and/or in the way the susceptible pool builds up. These are summarized in Table I. While all the three models permit the flow of susceptibles as newly born individuals in the population, the SIRS allows the surviving, infection-recovered individuals to join the susceptible pool after they lose their temporary immunity. The SEIR differs from the other two by introducing a lag period between the exposed and infectious stages while considering permanent protection from re-infection as in the SIR.

**TABLE I.** Components of internal factor controlling nonlinearity in three models of the SIR family.

Internal factor	SIR	SIRS	SEIR
Immunity	Life-long	Transient	Life-long
Lag from E to I	0	0	A few days

### B. Temporal forcing in transmission

The transmission rate is temporally modulated as follows:

$$\beta (t)=\beta_{0}(1+\delta F(t)).$$

The parameter *β*_0_ is the mean or unmodulated transmission rate. The parameter *δ* is the strength of seasonality that determines the amplitude of fluctuation in the transmission rate.^19^ The function *F*(*t*) determines the nature of modulation ranging from noisy, periodic, quasiperiodic, or chaotic. If the function has explicit dependence on time, then chaotic solutions may be observed.^20^ Such modulations have been studied in some detail in the dynamical systems context.^15,21,22^ In the epidemic modelling, random fluctuations in the transmission parameter were studied in Refs. 23 and 24. A simple sinusoidal function F(t)=sin t gives a periodically varying transmission rate.^5^ Since in this paper, we aim to study the effect of irregular temporal modulations in the transmission rate (i.e., *β* should temporally fluctuate with anomalies in its amplitude and/or onset of its peaks), we use a quasiperiodic function, F(t)=sin t+ϵ sin ωt, to temporally force the transmission rate, as follows:

$$\beta (t)=\beta_{0}(1+\delta (\text{sin}\;t+\epsilon \;\text{sin}\;\omega t)).$$ (4)

We consider the frequency in the above term as $\omega =\frac{\sqrt{5}-1}{2}$, it is the reciprocal of the golden mean,^16^ which is an irrational number. As one can see, ϵ is used to switch on and off the quasiperiodic term: ϵ = 0 recovers the periodic modulation while ϵ≠0 makes the transmission rate quasiperiodic. Here, we restrict the exploration of δ−ϵ space such that δ(1+ϵ)≤1 to keep β(t)≥0. Quasiperiodic modulation is known to create SNAs at the interface of regular and chaotic dynamics in the parameter space. Tori are the simplest regular dynamics possible in quasiperiodically modulated dynamical systems. SNAs have nonpositive largest Lyapunov exponents (implying no sensitive dependence of the dynamics on the initial conditions or starting values), but they are strange (i.e., they have nonsmooth/fractal geometry).^17^

**FIG. 1.**
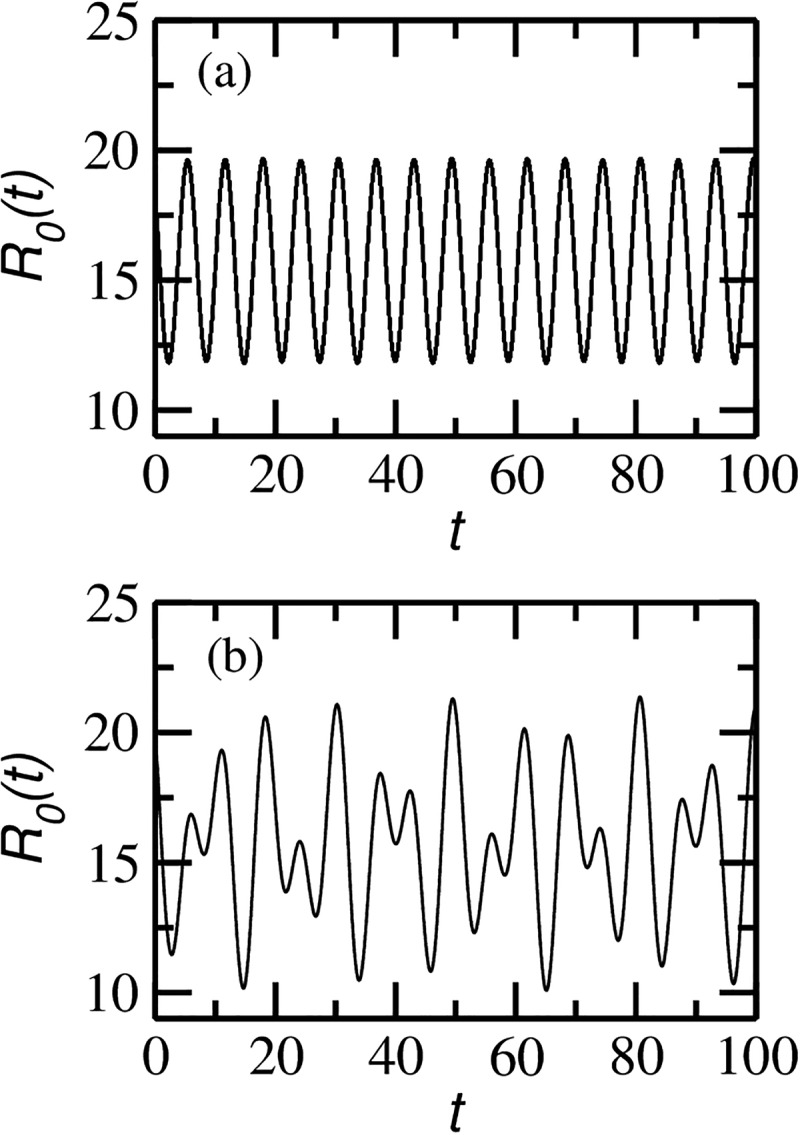
Time-dependent reproductive ratio $R_{0}(t)=\frac{\beta (t)}{\left(\mu +\eta \right)}$ as a function of time (see Eq. (4)) for two different *δ* and ϵ combinations: (a) δ=0.2471,ϵ=0.01 and (b) δ=0.184,ϵ=1.

The reproductive ratio is now time–dependent and is given by $R_{0}(t)=\beta (t)/(\mu +\eta )$. In a population a disease will eventually die out if the time average $\bar{R_{0}}(t)<1$. There can be however incidences of disease outbreak if $R_{0}(t)>1$ for some range of time. A time series representative of how the reproductive ratio $R_{0}(t)$ behaves for different combinations of ϵ and *δ* is shown in Fig. 1. The parameters chosen in Fig. 1 display strange nonchaotic dynamics for the SIR model to be discussed in Section III. In Fig. 1(a), the reproductive ratio looks like a sinusoidal time series while in Fig. 1(b) this resemblance is totally lost.

### C. Measures

In this subsection, we define three measures to distinguish the different types of attractors. We use the phase sensitivity $\frac{dX(t)}{dt}$^15^ to distinguish the fractal geometry of SNAs from that of smooth torus and chaotic motions. Here, *X*(*t*) represents the phase or state space of the attractor defined by the coordinates (S,I,E,R), depending on the model considered. In order to be able to capture the intermittent behaviour of phase sensitivity, one defines the following quantity:^15,16,25^

$$\gamma (L)=\underset{0\leq k\leq L}{\max}(|\frac{\partial X_{k}}{\partial t}|).$$ (5)

Note the partial derivative indicates the differentiation of any one coordinate or state variable (e.g., *S*(*t*)). Here, *L* represents a length of time. A growing γ(L) as a function of *L* is indicative of nonsmoothness of the underlying attractor.^15,16^ From γ(L), one can define the quantity

Γ(t)=min(γ(t,x,θ)), (6)

which is a collection of minima's over different initial points. For SNA $\Gamma (L)∼L^{q}$ *q* > 0, while for torus *q* = 0. For chaotic orbits $\Gamma (L)∼e^{qL}$. Hereafter, we call this quantity the phase sensitivity parameter. The phase sensitivity parameter shows linear growth for SNAs, an exponential growth for chaotic orbit, and non-growing behaviour for smooth torus,^15^ as captured by the exponent *q* along with the functional form.

Next, we introduce the mean square displacement $\left\langle r^{2}\right\rangle$.^26^ If *ψ* is any of the phase space coordinates (i.e., S, I, E, or R) and *ψ_ref_* is taken as a reference point, then the $\left\langle r^{2}\right\rangle$ is defined as follows:

$$\langle r^{2}\rangle =\frac{1}{L}\sum_{i=1}^{L}{\left(\psi_{i}-\psi_{ref}\right)}^{2}.$$ (7)

Here, we take $\psi_{i}=S(t_{i})$ instead of $\psi_{i}={\left[S(t_{i}),I(t_{i}),R(t_{i})\right]}^{T}$,^27^ where *t_i_* represents continuous time with i=1,2,...,L (we took *L* = 900 000) and $\psi_{ref}=0$. The $\left\langle r^{2}\right\rangle$ clusters around a single value for each coexisting attractor. Furthermore, this quantity converges rapidly with *L* (note *L* is the length of the time series) and thus provides a good measure in counting the number of coexisting attractors in addition to providing an estimation of basin sizes.

The Lyapunov exponents $\left\{\lambda_{i}\right\}$ $\left(\lambda_{1}>\lambda_{2}\ldots \right)$ are used to detect whether attractors are chaotic or nonchaotic.^28^ In particular, the largest and second largest Lyapunov exponents $\lambda_{1,2}$ have the following characteristics:

• For chaotic orbits, $\lambda_{1}>0$.
• For limit cycles, $\lambda_{1}=0$ and $\lambda_{2},\ldots ,n<0$.
• For stable fixed points, $\lambda_{i}<0$.
• For smooth torus, $\lambda_{1}=\lambda_{2}=0$.
• For SNA, $\lambda_{1}=0,\lambda_{2}<0$, although the phase sensitivity parameter is able to distinguish between SNA and other orbits.^15^

The criterion of the largest Lyapunov exponent (LLE) for SNA in maps is that the largest nonzero Lyapunov exponent must be negative, while for flows it has to be zero. In the results shown in this work, we are able to ignore the zero Lyapunov exponent (for flow one, LE must always be zero) due to time coming explicitly in the dynamical equations Eq. (4).

The evolution of the basin of attraction of SNAs can be studied using the distribution of Lyapunov exponents in the space of initial conditions. For different initial conditions, if the largest non-zero Lyapunov exponent clusters around a single value then one can say, that those initial conditions belong to the basin of attraction of the same attractor.

### D. Model parameters and numerical simulations

For all the results provided below, unless otherwise stated, we set $\beta_{0}=1575$ (yr)^−1^, *η* = 100 (yr)^−1^, and σ=1/0.0279 (yr)^−1^, along with μ=0.02 (yr)^−1^. The disease parameter values are typical of measles infection dynamics and are taken from Ref. 29. The dynamics of the SIRS model is explored for a set of values (in yr^−1^) of the parameter *κ* and are provided where applicable. We integrate the system dynamics numerically using the fixed-step fourth-order Runge–Kutta method^30^ with a time step size of 0.001, including the calculations of the Lyapunov exponents and other measures described in Section II C. Initial conditions are provided at appropriate places in the text.

## III. RESULTS AND DISCUSSION

First, we consider the SIR model. Here, the condition for infectives to grow gives a threshold for susceptibles $S>1/R_{0}(t)$, where $R_{0}(t)$ is the reproductive ratio.^2,5^ Numerical integration of the model Eq. (1) gives information about how S(t) and I(t) change and infection outbreak occurs with time. The dynamics of epidemic models under periodic modulation of the transmission rate (i.e., ϵ=0,δ≠0) has been a subject of extensive study.^1–3^ By introducing periodic modulation in the transmission rate, infection dynamics displays a repertoire of periodic to chaotic behaviours. The route to chaos is period doubling. In the periodic modulation case, there is infinite period doubling, whereas in quasiperiodic modulation finite doubling of torus before the onset of chaos.^15^

**FIG. 2.**
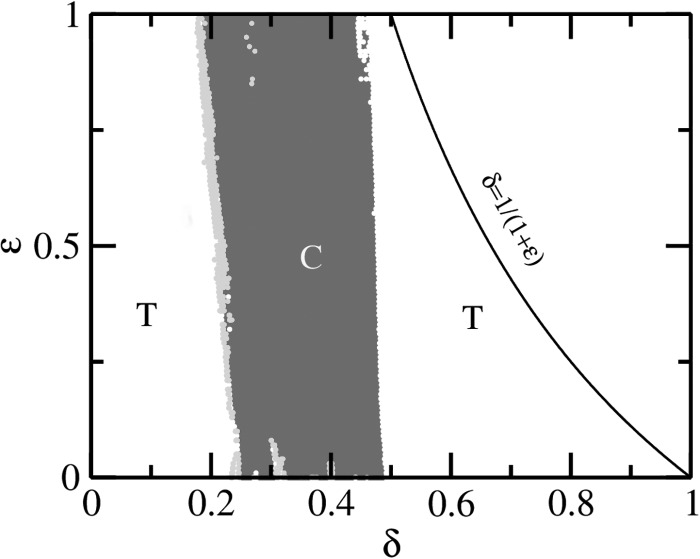
The *δ*–ϵ parameter space for the quasiperiodically forced SIR model. The red regions are of chaotic (C) dynamics while white indicates periodic or regular torus (T) dynamics. The strange nonchaotic attractors lie at the regular–chaos boundary (green). These regions were demarcated using the largest nonzero Lyapunov exponent.

The organization of the dynamics in the δ−ϵ parameter space is shown in Fig. 2. To obtain the parameter space, we choose for each ϵ the initial conditions $S_{ic}=0.1,I_{ic}=0.08,R_{ic}=1-S_{ic}-I_{ic}$ at *δ* = 0, and then using the last coordinate values of the orbit obtained as the initial conditions for next *δ*, separated by 0.001. We calculate the largest nonzero Lyapunov exponent (LLE) of the system and choose the color red wherever LLE is positive and the color white whenever it is zero. The SNA dynamics, which occur at the boundary of the regular and chaotic dynamics, are characterized by the green color. Note that the line ϵ = 0 corresponds to the periodic modulation while nonzero ϵ lines, each separated by 0.001 from next, represent the quasiperiodic modulation.

**FIG. 3.**
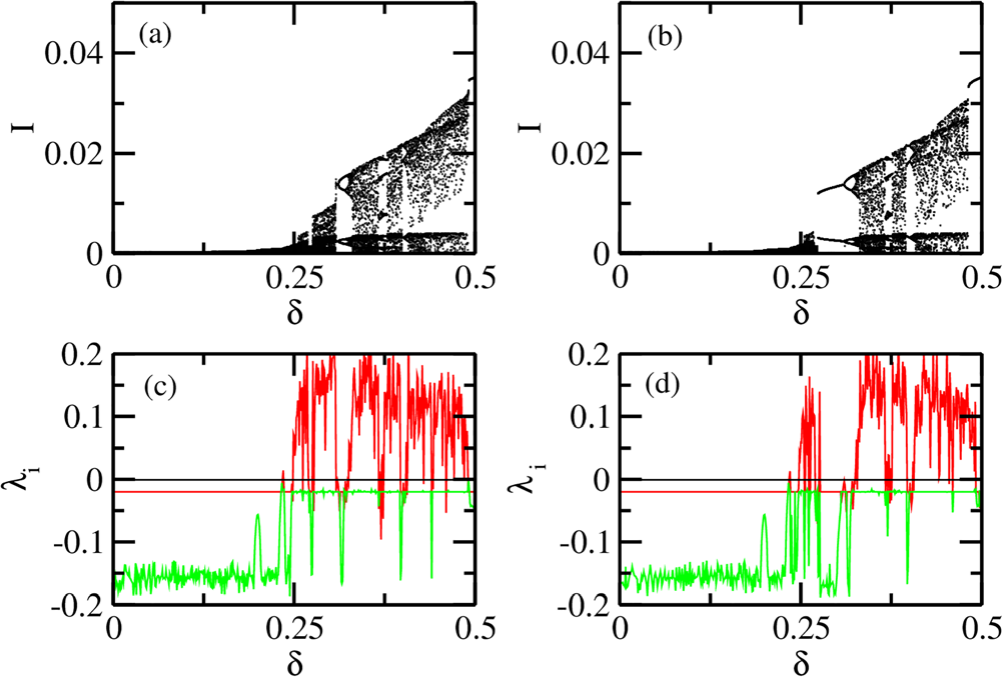
The coexistence of multiple attractors in terms of long-term dynamics of the periodically forced SIR model. Bifurcation diagrams are shown as a function of modulation parameter *δ* in Eq. (4) with ϵ=0.0 in the forward (a) and backward (b) directions. The corresponding two nonzero largest Lyapunov exponents (red and green) are shown in (c) and (d). The coexistence or multistability of attractors can be observed from the bifurcation diagrams as well as the Lyapunov exponents.

Fig. 3 shows the coexistence of multiple attractors for the periodic modulation of the transmission rate in terms of forward (Fig. 3(a)) and backward (Fig. 3(b)) bifurcation diagrams and the corresponding Lyapunov spectra for forward (Fig. 3(c)) and backward (Fig. 3(d)) bifurcations. When the dynamics is in the periodic regime (e.g., for small *δ*), the frequency of oscillations is the same or an integral multiple of the forcing frequency (taken to be unity here). However, when *δ* is increased, chaotic dynamics is achieved via period doubling route.^20^ In the chaotic regime, unpredictable episodes of epidemic outbreaks can occur over time. The forward bifurcation and the corresponding Lyapunov spectrum were obtained by choosing a set of the initial conditions at *δ* = 0 and then using the coordinates of the orbit obtained as the initial conditions for next *δ*, separated by 0.001. A similar procedure was adopted for the backward bifurcation, beginning at δ=1/(1+ϵ) and moving towards *δ* = 0 in steps of 0.001. This procedure of deriving the attractor types is called the continuation of attractor. It ignores the multiple coexisting attractors^5^ while traversing in either direction. However, the coexistence is revealed by comparing the attractors constructed by both the forward and backward procedures.

**FIG. 4.**
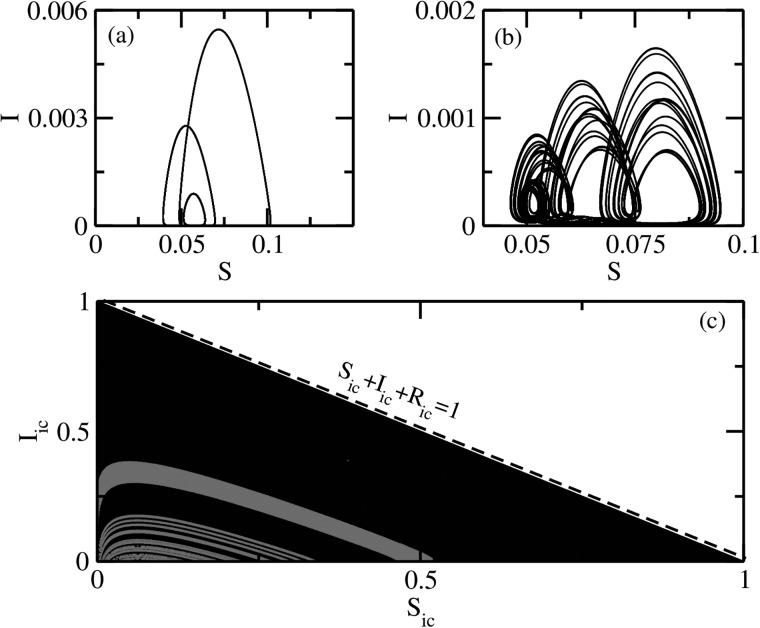
Coexisting periodic and chaotic attractors for the periodically forced SIR model and their basins: (a) periodic attractor, (b) chaotic attractor, and (c) the basins belonging to two distinct attractors–the red regions are the basin of attraction for the attractor in (a) while the black regions the basin of attraction of the attractor in (b). Here, the parameters are fixed at δ=0.25 with ϵ = 0 (only periodic modulation), μ=0.02, and *η* = 100.

As reported in previous studies,^5,10,18,19^ an important feature of the periodically forced SIR system is the existence of multistability where two different attractors may coexist. For one such parameter value, the coexisting attractors are shown in Figs. 4(a) and 4(b) along with their basin of attraction in Fig. 4(c). To obtain the basins, we used the largest nonzero Lyapunov exponent and the mean square displacement (Eq. (7)) as indicators of orbits. These orbits were obtained by varying the initial conditions in the *S_ic_*–*I_ic_* plane with *R_ic_* being determined by the constraint $S_{ic}+I_{ic}+R_{ic}=1$. Clearly, the relevant initial conditions in the *S_ic_*–*I_ic_* plane are limited to a triangular region with its vertices at (0,0) (1,0), and (0,1). The coexistence of multistability implies that different initial states may result in different asymptotic states even if we keep all other parameters fixed.

### A. Endemic dynamics under quasiperiodic forcing

**FIG. 5.**
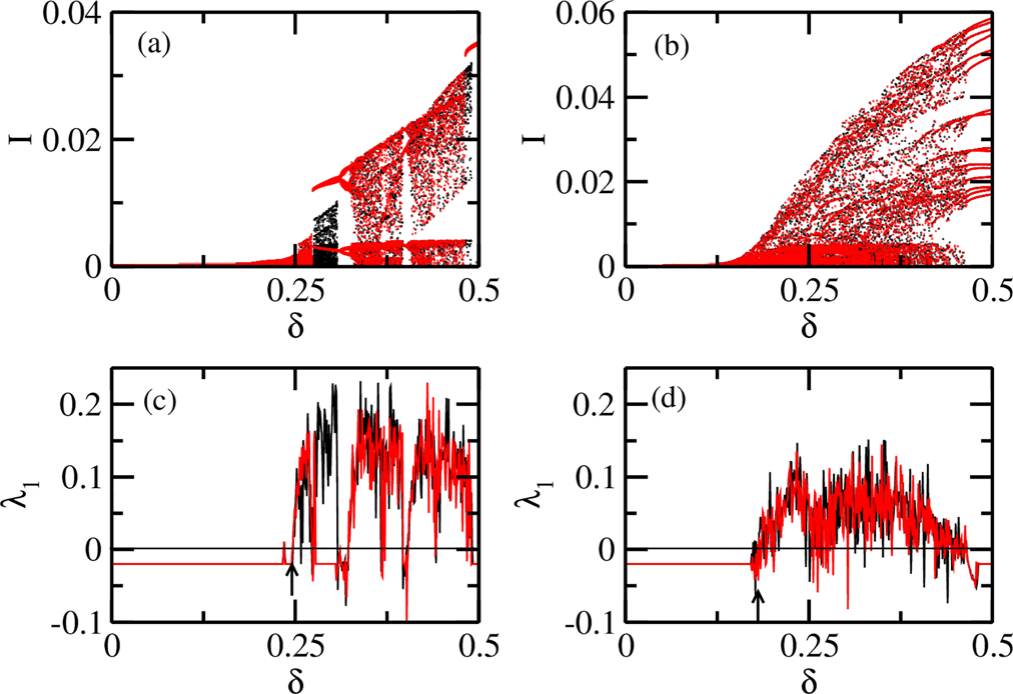
Long-term dynamics of the quasiperiodically forced SIR model. Bifurcation diagrams are shown as a function of *δ* in Eq. (4) with ϵ=0.01 (a) and ϵ = 1 (b). The corresponding largest nonzero Lyapunov exponents are shown in (c) and (d). The data shown in black and red, respectively, were obtained by the forward and backward procedures. The boxes formed by the difference in the forward and backward Lyapunov exponent indicate multistability, some of which can be seen from the bifurcation diagrams as well. Clearly there are no indication of multistability at ϵ = 1. The little arrows in (c) and (d) indicate the parameter values around which SNAs are found (see Figs. 6 and 8).

**FIG. 6.**
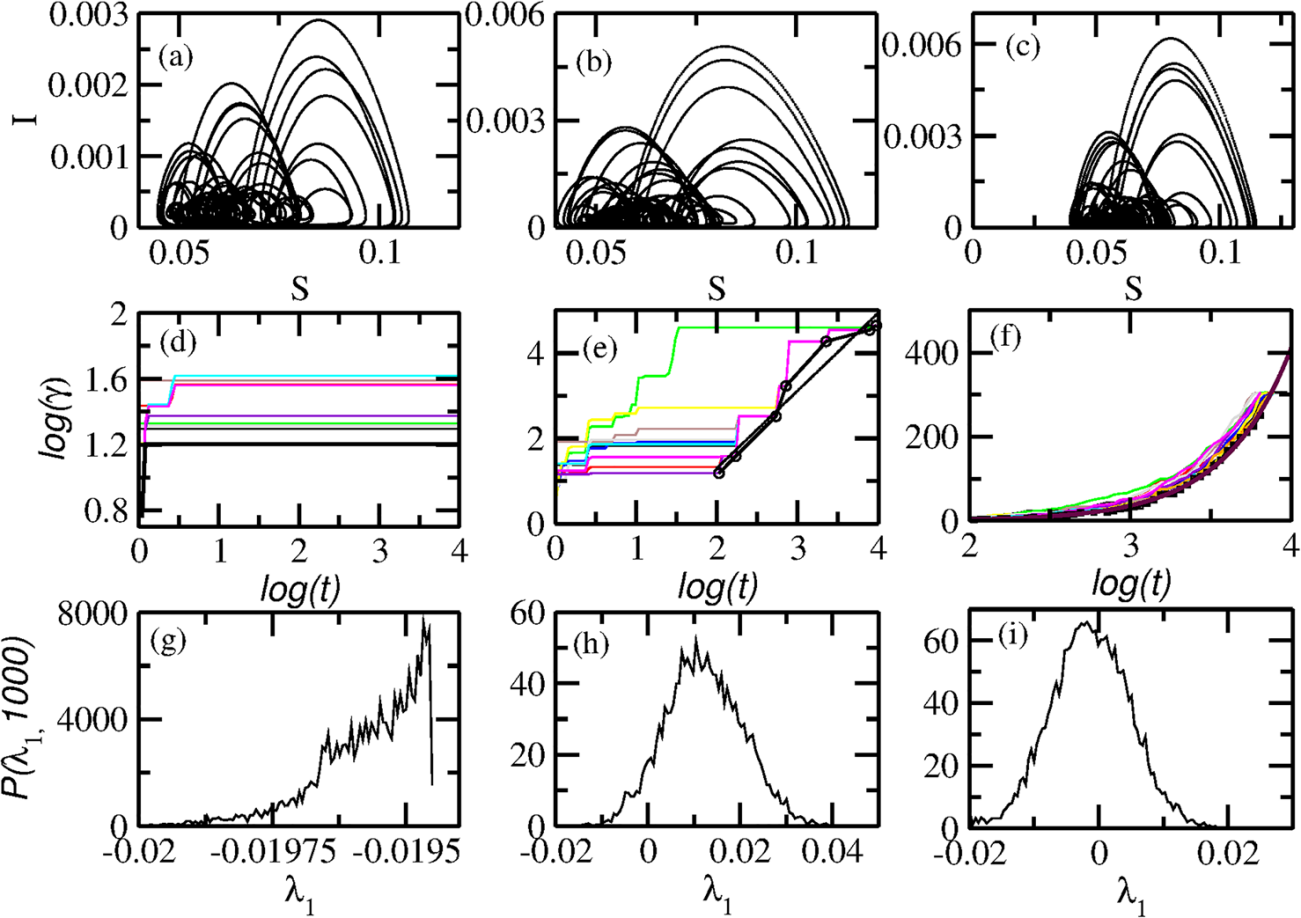
Three types of dynamics in the quasiperiodic SIR model. The trajectory types for ϵ = 1 and different *δ* values are as follows: (a) a regular torus at δ=0.165, (b) an SNA at δ=0.18, and (c) a chaotic orbit at δ=0.184. The plots (d)–(f) show the respective phase sensitivity parameter, over different initial conditions, as a function of trajectory length showing that the trajectory in (a) is smooth while those shown in (b) and (c) are non-smooth. The plots (g)–(i) show the distribution of finite time largest Lyapunov exponents, $P(\lambda_{1},t)$ with *t* = 1000. The sensitivity exponent defined in Eq. (6) is *q* = 0 for the torus in (a) and q∼1.82 for SNA in (b) as obtained by curve fitting. The chaotic orbit shows a growth of the form $\Gamma (t)∼e^{qt}$ with q∼2.43. The curve for Γ(t) along with best fits is shown in black.

When the transmission rate is quasiperiodically modulated (i.e., ϵ≠0), the simplest nonchaotic dynamics happens on a smooth torus surface.^15^ This implies that the susceptible (or infectious, etc.) fraction varies with an irrational frequency (because forcing frequency is irrational). Consequently, it cannot be claimed that the peak in disease incidence would repeat itself periodically in time, as happens in the periodic modulation case. On increasing the values of *δ*, the dynamics changes from torus to chaos via a finite period doubling route.^15^ Strange non-chaotic attractors may be observed at the transition boundary between torus and chaos. A typical feature of SNA is that the dynamics are free from sensitive dependence on initial conditions: two trajectories started with slightly different initial conditions synchronize on the SNA^31^ but have non-smooth or fractal geometry. This implies that asymptotically predictable pattern of epidemic outbreaks is possible under quasiperiodic forcing. This is because a SNA trajectory has both contracting and expanding subsets of the attractor and for a sufficiently long trajectory the contracting set dominates, rendering the dynamics free from sensitive dependence on initial conditions. The SNAs are observed at the transition boundary of chaotic and regular motions as one varies *δ*, see the green regions in the two dimensional parameter space shown in Fig. 2. Examples of the typical bifurcation diagram as a function of *δ* for different values of ϵ are shown in Figs. 5(a) and 5(b), both containing the forward (black dots) and backward (red dots) bifurcation plots. Although the bifurcation diagrams for the quasiperiodic modulation case are shown, it is important to note that the dynamics occurs on a quasiperiodic torus and the standard bifurcation theory does not apply: n–branch orbits are converted to $T^{n}$, etc. The corresponding Lyapunov exponent spectra are shown in Figs. 5(c) and 5(d). Orbits are distinguished from each other based on the Lyapunov exponents (see Section II C) and the phase sensitivity parameter described in Eq. (5). Typical orbits in the torus, SNA, and chaotic regimes are shown in Figs. 6(a)–6(c), their corresponding phase sensitivity parameters in Figs. 6(d)–6(f), while the distribution of finite time largest Lyapunov exponents, $P(\lambda_{1},t)$ with *t* = 1000, are shown in Figs. 6(g)–6(i).

**FIG. 7.**
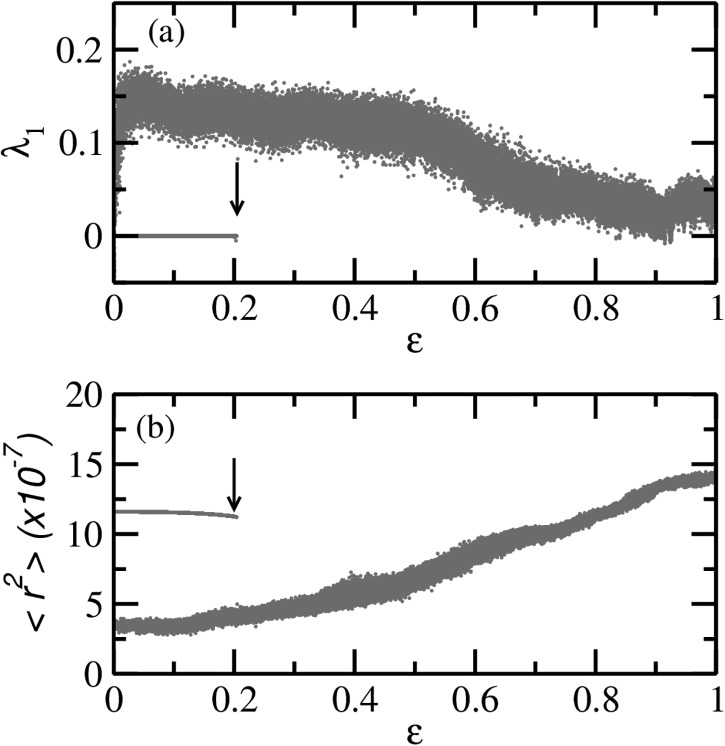
(a) The largest nonzero Lyapunov exponent and (b) the mean square displacement $\left\langle r^{2}\right\rangle$ as a function of ϵ at δ=0.277 obtained for the dynamics of the quasiperiodically forced SIR model. Note that both diagrams indicate that multistability disappears after a critical ϵ_*c*_ marked by black arrows.

**FIG. 8.**
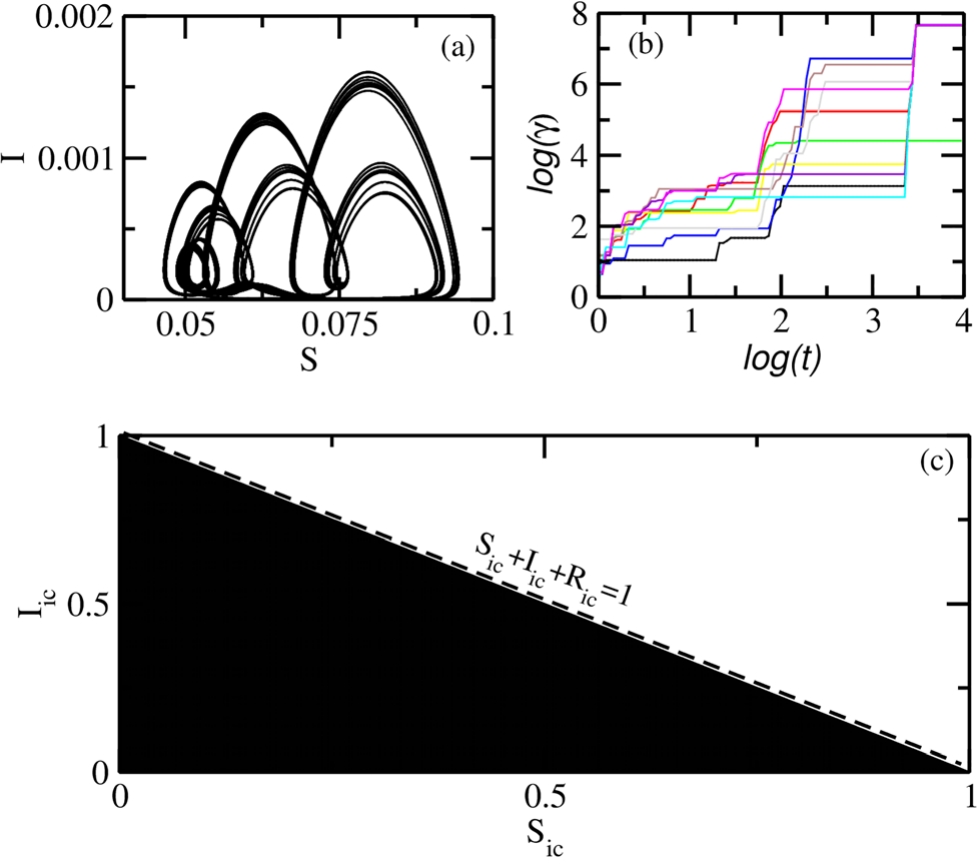
(a) SNA observed at δ=0.2471,ϵ=0.01 in the quasiperiodically forced SIR model. (b) Its phase sensitivity parameter over different initial conditions, and (c) basin of attraction, indicating that no other orbits coexist with it at this point in the parameter space. The other parameters are fixed at μ=0.02,η=100.

Since multistability is observed in the periodically modulated case, it is natural to check the fate of coexisting attractors and their basins under quasiperiodic modulation. To this end, we look for multiple attractors using the mean square displacement and the largest nonzero Lyapunov exponent (see Section II C), calculated for a set of 50 initial conditions on the line $I_{ic}=S_{ic}/2000$ by fixing *δ* and varying ϵ. We find that multistability disappears after a critical value ϵ_*c*_ is reached. The critical value ϵ_*c*_ is different for different *δ*. A typical example for δ=0.277 is shown in Fig. 7: the largest nonzero Lyapunov exponent in Fig. 7(a) and mean–square displacement in Fig. 7(b). Distinct clusters of the largest nonzero Lyapunov exponents or the $\left\langle r^{2}\right\rangle$ indicate the coexistence of distinct attractors. Now that we know a critical value of ϵ after which only one attractor survives for a given *δ*, we choose a value of $\epsilon <\epsilon_{c}$ for which it is expected to see multistability as we increase *δ* from zero. We stop at a value of *δ* at which an SNA is likely to be observed and scan for the other coexisting attractors and their relative basin sizes. We find that typically an SNA occupies the whole basin: Fig. 8(a) shows the SNA at δ=0.2471,ϵ=0.01, Fig. 8(b) its phase sensitivity parameter, and Fig. 8(c) shows its basin.

The SIRS and the SEIR models behave like the SIR model when κ→0 or σ→∞, respectively. In these limits, the analysis of SIRS and SEIR does not differ from that of the SIR model. Below, therefore, we present results for these two models when *κ*/*σ* is in the regime which makes them distinct from the SIR model in the *δ*–ϵ space.^32^

**FIG. 9.**
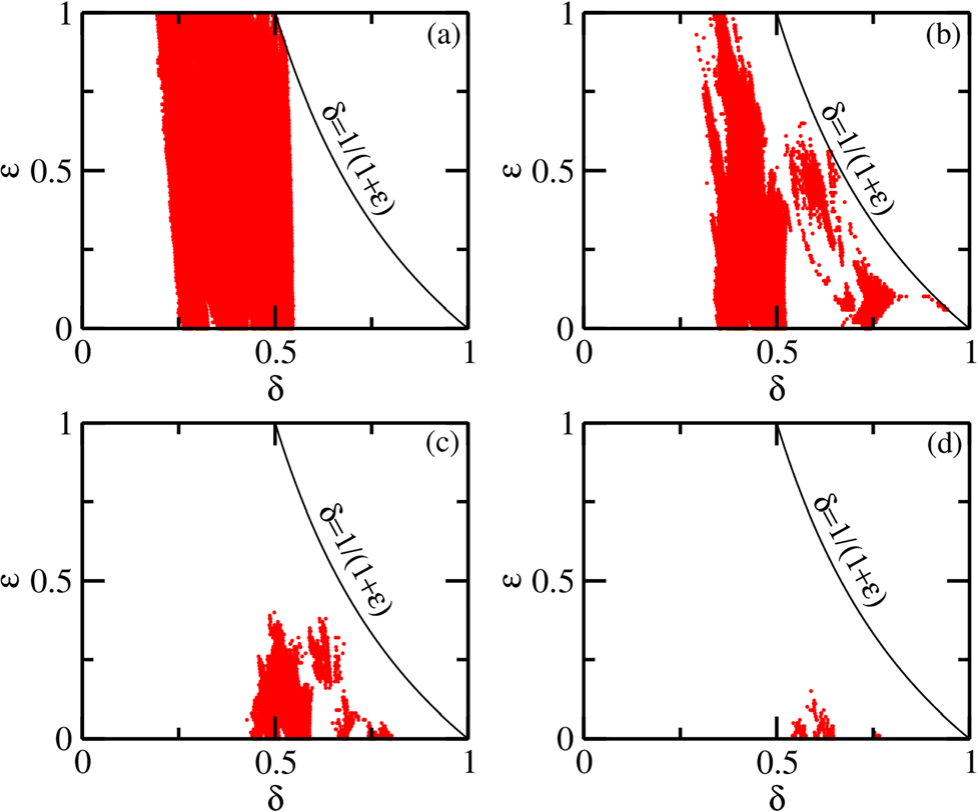
The *δ*–ϵ parameter space for the quasiperiodically forced SIRS model. The red regions are of chaotic dynamics while the white region indicates torus or periodic dynamics. The strange nonchaotic attractors lie at the regular–chaos boundary (not shown). These regions were demarcated using the largest nonzero Lyapunov exponent. The chaotic regions decrease as *κ* is changed from (a) 0.001, (b) 0.01, (c) 0.02, and (d) 0.03.

In the SIRS model, if the immunity loss rate *κ* is sufficiently high then the chaotic behaviour is reduced to very narrow ranges of *δ* for the periodically modulated case (ϵ = 0). When quasiperiodic modulation is switched on (ϵ≠0), the chaotic regions are further reduced, eventually disappearing altogether above a certain ϵ as shown in Figs. 9(a)–9(d).

**FIG. 10.**
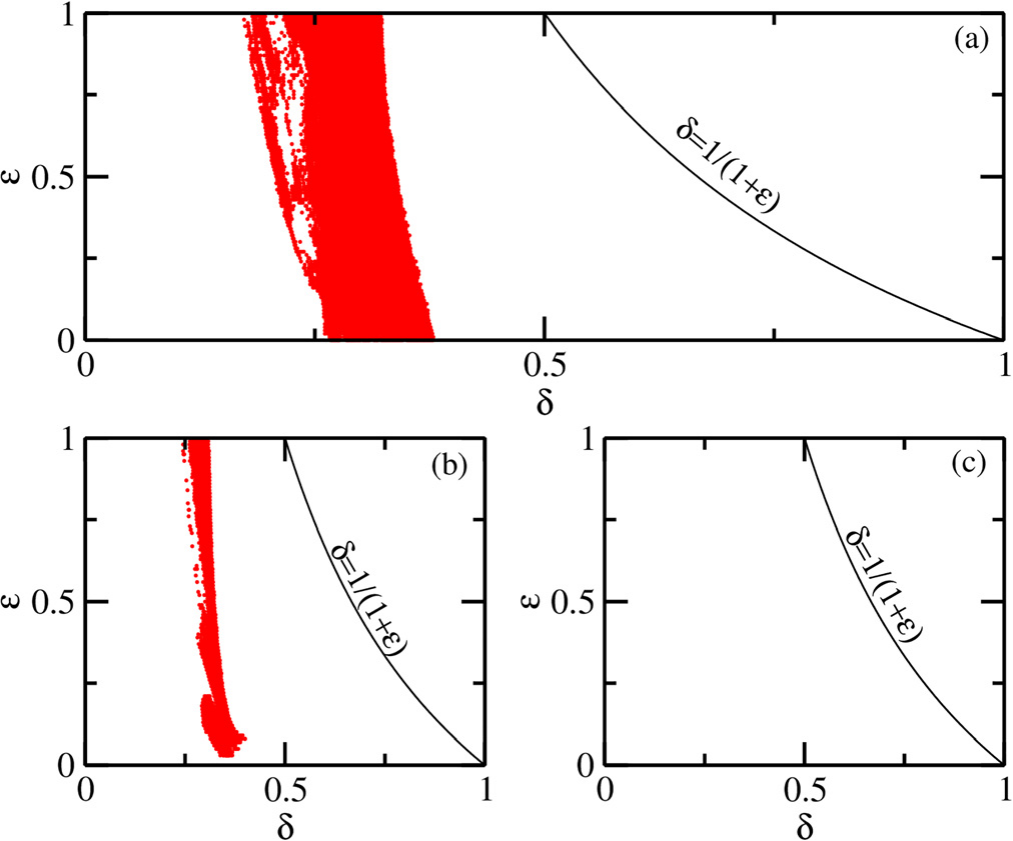
The *δ*–ϵ parameter space for the quasiperiodically forced SEIR model. The red regions are of chaotic dynamics while the white region indicates torus or periodic dynamics. The strange nonchaotic attractors lie at the regular–chaos boundary (not shown). These regions were demarcated using the largest nonzero Lyapunov exponent. The parameter *σ* varies from (a) 35.8423, (b) 15.0, and (c) 8.0. The chaotic regions gradually become narrower as *σ* is decreased.

In the periodically modulated (ϵ = 0) SEIR model, we find that as σ→0 the chaotic region is reduced to narrow ranges of *δ* before finally disappearing below *σ* = 10 (see Figs. 10(a)–10(c)). Thus, when the latent period is large, even the periodic modulation of the transmission parameter in the SEIR model is not capable of generating complicated dynamics beyond periodic orbits as σ→0. However, if for some *σ* chaotic dynamics is observed for a finite range of *δ* then, unlike the SIRS model, the introduction of quasiperiodic modulation ϵ≠0 does not eliminate chaotic regions in the *δ*–ϵ space.

The reduction in chaotic regions in the SIRS and SEIR models may be understood from the following arguments: recall that as κ→0 (recovered individuals do not become susceptible again) and σ→∞ (the latent period tends to zero) reduce the SIRS and SEIR models, respectively, to SIR. Then, taking the opposite limit κ→∞ would imply the individuals are less likely to stay in the recovered state thereby reducing the dimension of the dynamical equations to two with only S and I as effective variables. Similarly, σ→0 would mean that susceptible individuals never get to the infective state, thus once again reducing the dimension of the dynamical equation to two with only *S* and *E*. It is known that only three or higher dimensional autonomous flows display chaotic behaviour. Thus, the result of our numerical experiments that chaotic regions start depleting as soon as we increase *κ* or decrease *σ* is due to the lower dimensional dynamics taking over even for *κ* and sigma being far from that absolute limit.

## IV. SUMMARY

Previous studies, investigating the effects of periodic modulation in the transmission rate, showed the coexistence of multiple attractors in the dynamics of the SIR family of epidemic models. They helped in understanding important implications of seasonality for transmission ecology, dynamics, and control (e.g., by vaccination) of infectious diseases.^1–3,10,18,19^ However, these studies ignored the impact of variability in the external driving signals and that of internal factors (e.g., immune responses) as the source of nonlinearity in disease dynamics itself on the infection dynamics and coexistence of multiple attractors. This exclusion is increasingly surfacing as a significant element to be considered in disease modelling (see the arguments put forth in Refs. 6 and 7) as we progressively gather empirical evidence that the external driving signals (i.e., rainfall, temperature, etc.) may indeed vary year-to-year^11,12^ and therefore their impact may not be captured by a sinusoidal wave function.

In this paper, we studied the effects of quasiperiodic modulation in the transmission rate in the SIR and its allied models such as the SIRS and SEIR. In physics and engineering sciences, there have been extensive studies in forced oscillator systems using different forcing functions, including quasiperiodic one, and this study is a first attempt, as far as we are aware of, to apply and investigate the effect of quasiperiodic forcing of the transmission terms in epidemic models. The addition of quasiperiodic element in the temporal modulation of the transmission term gradually (as ϵ increases) annihilates multistability, leaving behind only one attractor for each parameter set. Additionally, new dynamical states, such as the SNAs, are created which make the dynamics of epidemics asymptotically predictable although they have non-smooth geometry (chaotic states too have non-smooth geometry). However, in the SNA states there can be unpredictability in outbreaks of disease in finite time.

The coexistence of multiple attractors (in the periodically modulated case), such as chaos and periodic orbits, is believed to provide an explanation for observed different trajectories of the incidence of childhood diseases (e.g., measles) in the post-vaccination era from those dominated in the pre-vaccination era.^2,18^ The coexistence of multiple attractors (such as chaotic and smooth torus) is still likely as in the periodically forced SIR models albeit for smaller values of ϵ. However, when the disease dynamics are dominated by the SNA-type trajectories, we find that it occupies the whole basin and no other states coexist. The existence of SNAs provides an additional state available where the dynamics is asymptotically predictable.

Since SNAs in epidemic models created under quasiperiodic forcing are not sensitively dependent on initial conditions (as they occupy the whole basin), control efforts such as vaccination may not be able to alter the predictability of disease incidences. Periodically forced SIR dynamics were hypothesized to be able to switch and settle on different attractors in the presence of noise,^19^ we hypothesize that noise-induced switching of trajectories may not be apparent due to the presence of SNAs in quasiperiodic forced epidemic models. In addition, the SIR models considered here are simple in comparison to the models of vector-borne or environment-mediated infectious diseases (such as malaria and cholera, respectively). Year-to-year variation in external forcing factors is likely to introduce temporal heterogeneity in the persistence and density of disease vectors or causative agents (e.g., *Vibrio cholerae*) in the environment, and this effect will be more pronounced in the regions of marginal environmental conditions.^7^ Therefore, the dynamics of more complex disease models under quasiperiodic forcing of the transmission rate will be highly relevant. We plan to investigate these questions in future.

Regarding the problem of persistence in metapopulations, it is known that seasonal forcing of transmission has the tendency to synchronize epidemics among sub–populations.^2,33^ In uncoupled metapopulations, chaotic dynamics, however, does not synchronize although the strange nonchaotic attractors, which are created when transmission rate is quasiperiodically modulated, can synchronize. An investigation of metapopulations when transmission is quasiperiodically modulated will form the basis of our future work.
